# Scientific and Ethical Issues in Exporting Welfare Findings to Different Animal Subpopulations: The Case of Semi-Captive Elephants Involved in Animal-Visitor Interactions (AVI) in South Africa

**DOI:** 10.3390/ani9100831

**Published:** 2019-10-19

**Authors:** Barbara de Mori, Elena Stagni, Linda Ferrante, Gregory Vogt, Keith A. Ramsay, Simona Normando

**Affiliations:** 1 Department of Comparative Biomedicine and Food Science, Università degli Studi di Padova, viale dell’Università 16, Agripolis, 35020 Legnaro PD, Italy; barbara.demori@unipd.it (B.d.M.); linda.ferrante@phd.unipd.it (L.F.); 2 Ethics Laboratory for Veterinary Medicine, Conservation, and Animal Welfare, Università degli Studi di Padova, viale dell’Università 16, Agripolis, 35020 Legnaro PD, Italy; estagni.res@gmail.com (E.S.); greg@conservationguardians.co.za (G.V.); 3 Independent researcher, Via Ranzani, 17, 40127 Bologna, Italy; 4 Conservation Guardians, Shongweni Nature Reserve, Outer West, 3610 Kwa Zulu Natal, South Africa; 5 Department of Agriculture Forestry and Fisheries, South Africa—former Scientific Manager, Animal Production, 20 Steve Biko former (Beatrix) Street, Agriculture Place, Arcadia 0031, Pretoria, South Africa; keithrms9@gmail.com

**Keywords:** animal welfare, elephants, ethics, external validity, assessment, animal-visitors interactions

## Abstract

**Simple Summary:**

In southern Africa, several elephants are involved in ‘wildlife tourism interactions’ with tourists, whose acceptability is the focus of much media interest. It is important that the welfare of the animals involved in such activities is monitored in order to grant them an acceptable quality of life. Until now, protocols to assess welfare in African elephants have been developed only for zoo elephants. However, protocols developed for a different situation may not be suitable for these elephants, which live under different circumstances (for example, in some cases they tend to be able to roam free in the bush for a part of the day and to be allowed contact without protective barriers with people). We discuss the possible problem of extending findings found in zoo elephants to elephants involved in activities with tourists outside the zoos. This concern was also highlighted by elephant experts who said that in 23.6% of cases the main welfare problems of zoos’ elephants were different from those of elephants involved in interactions with tourists in South Africa. Moreover, their agreement was low when they were asked the acceptability of some procedures, which are often applied differently in zoos and in the facilities offering interactions with tourists.

**Abstract:**

Elephants are charismatic, cognitively highly-developed animals, whose management conditions can vary along a “wild–captive continuum.” Several protocols have been proposed for the assessment of zoo elephants’ welfare. It is important to investigate the possible limitations, if any, of extending findings from zoo elephants to conspecifics in a different dynamic in said “wild–captive continuum.” In this paper, findings regarding two issues will be discussed: those regarding the external validity and those regarding the acceptability of management procedures as applied to semi-captive (i.e., able to roam freely for part of the day) elephants involved in visitor-interaction programs in South Africa. In a questionnaire-based survey, half of the responding experts stated that at least some of the welfare issues they ranked as the five most important in captive elephants’ management had a different relevance for semi-captive individuals, resulting in 23.6% of the issues being rated differently. Moreover, there was no agreement among the experts on the ethical acceptability of any of the investigated procedures used in the management of semi-captive elephants involved in visitor-interaction programs. Caution is thus needed when exporting findings from one subpopulation of animals to another kept in different conditions and more scientific and ethical research is needed on the topic.

## 1. Introduction

African elephants (*Loxodonta africana*) are highly charismatic animals, who have a rich social life [1,2,3] and complex cognitive abilities [4,5,6,7,8,9]. Therefore, it is not surprising that their welfare in captive conditions has been the focus of much scientific interest, such as that of primates [10,11,12]. Like primates, elephants under human care can be described as being distributed on a “wild–captive continuum” in different situations, as outlined by Hosey [13] for primates. However, most of the research on elephant welfare has been done on zoo animals. Hence it is important to reflect on the possible risks of transferring findings from zoo animals to elephants on a different position in the “wild–captive continuum.” The risk of underestimating existing differences in the range of management options is high. Animal welfare is highly influenced by the context in which animals are housed and by choices about how to manage them: different management methods in the “wild–captive continuum” involve not only different housing conditions, but also different stakeholders and different values. An example can be the so-called “semi-captive” elephants housed in facilities that offer a range of interactions between the elephants and tourists in South Africa. It is worth noting that, while most of the scientific studies proposing methods/parameters to assess welfare include various reliability checks (e.g., concordance among different observers, and concordance between data taken some time apart), as reviewed by Williams et al. [14], for elephants, external validity is virtually always ignored. The same is true for a comparative study about ethical acceptability of management options.

This paper is part of a line of research aimed at studying subpopulations of wild animals which are involved in visitor-interaction programs both in zoos and other facilities offering such interactions, by merging a scientific and an ethical approach. Such subpopulations are managed in a way that is often slightly different from other captive subpopulations, if not for other reasons, at least for the fact that they are often asked to have a direct interaction with visitors in “free contact” conditions (see below). It is therefore important to analyze both the scientifically and the ethically relevant aspects of such differences, mainly because they can be relevant when assessing as well as when attempting to improve the welfare of these animals. The aim of this paper is to highlight potentially welfare-relevant differences between semi-captive visitor-interacting elephants in South Africa and zoo elephants in order to stimulate debate about the pros and cons of exporting welfare assessment methods from one subpopulation of a wild animal species to another kept in different conditions.

It is now widely accepted that science and ethics are inextricably intertwined facets of animal welfare’s evaluation [15,16]. Ethics, together with economics and social sciences, plays a role both in the definition of animal welfare [17,18] and in decisions about the minimum acceptable level of welfare to provide [19,20] and dictates the acceptability of the animal’s life conditions. Ethics, therefore, play a role in the designing of protocols addressing and evaluating welfare issues [21]. Ethics is also needed to assess specific, controversial welfare-relevant issues, which can vary depending on the animal population studied. In this study, we discuss both the issue of external validity per se and that of acceptability of management procedures that are typical of semi-captive animal-visitor interactions.

### The “Semi-Captive” Elephants in South Africa

Facilities offering interactions with animals belonging to wild species to tourists, similar to those in Thailand [22], are widespread in southern Africa. For example, in South Africa, around 120 elephants [23] are kept in facilities defined as captive facilities [24], but which fulfil the criteria we use to define semi-captive animals. These facilities are open to the public who pay a fee to enter them. The common ground of these facilities is that they offer some form of animal–visitor interaction with the elephants they house. The interactions offered by these captive elephant facilities range from attending training shows, to hand feeding, walking with, and even riding on the elephants. Usually, these interactions include forms of non-protected contact between people (both handlers and public) and elephants [25], whereas a policy of “protected contact” has long been recommended for western zoos [26]. In the protected contact system, human and animal spaces are separated; the elephants are managed using positive reinforcement training and punishment is not allowed. Trainers never attempt to socially dominate the elephants [27]. These facilities can be extremely different from one another in their organization and the way they function, and each facility represents a range of management dynamics. However, there are some common traits: Apart from being involved in interactions with people, as already mentioned, the animals kept in most of these facilities tend to be somewhat less space-restrained than zoo-housed elephants, at least for part of the day. For example, some of them live on large tracts of land and are lured by high-energy food rewards to come in for interactions. Others are stabled in pens and/or buildings and are herded to the bush for part of the non-show/interaction time. Once in the bush, the elephants are let free to choose what to do (which is usually foraging) and to move around, without any imposed activity, under the supervision of their handlers, unless there is some emergency prompting the handlers to intervene. This virtually non-restricted activity in the bush will be referred to as free choice activity, (FCA). The ability to explore and interact in a virtually non-restricted way with the natural environment is deemed to be an important, welfare relevant, cognitive stimulant for animals [28]. Clubb and Mason [29] describe the system of working Asian elephants—tamed and trained elephants not held in zoos—as extensive. The authors cite agricultural literature, which uses ‘extensive’ in contrast to ‘intensive,’ to indicate a less physical restraint [30]. The use of this term might be linked to the fact that during the night the Asian working elephants are allowed to move in forests, although hobbled, and to feed on natural vegetation [31]. Groups are large and mixed, and encounters with wild elephants are common [32]. It can be argued that the greater distances roamed, the possibility to select the food and to show more natural behaviors have a positive influence on the elephants in extensive or semi-captive conditions [33]. In the South African facilities, the elephants are generally confined into paddocks or stables during the night, but most have a certain degree of freedom during the day, being herded around the reserve for FCA for a variable time of the day. It is, therefore, reasonable to define them as semi-captive, or as extensively managed as Asian working elephants are, differences notwithstanding. It is important to note though, that whilst most of these elephants are indeed less restrained than their zoo counterparts, there could be exceptions. In some cases, they could be released only in a fenced area and called back to the stable for the night, or, sometimes when the “interaction with tourist” agenda is full, they could be allowed to roam for shorter periods of time in a paddock close to the stables.

It is, therefore, logical that when assessing the welfare of such elephants, one needs to address both their distinctive human-controlled general management and the specific issues related to the interactions with visitors.

## 2. The Issue of External Validity

One of the main scientific issues in welfare evaluation is the overall quality and meaning of the parameters used in the assessment. Scientific evaluation relies on measurements that have to be reliable and valid [34]. “Reliability concerns the extent to which measurement is repeatable and consistent: that is, free from random errors.” [34] (p. 72). “Validity concerns the extent to which a measurement actually measures what the investigator wishes to measure and provides information that is relevant to the question being asked.” [34] (p. 73). Validity has often been a major source of concern, as the biological meaning of the measures is seldom known, and many parameters tend to be unspecific (e.g., changes can be elicited by both positive and negative arousing experiences [35]). Another important facet of validity, which can be important in animal welfare research, is that of “external validity,” defined as the extent to which the results are applicable “to other situations (environmental contexts), population, or species” [36] (p. 124). This aspect is particularly relevant when considering the welfare of individuals of wild animal species under human management, because the different ways they are managed can be seen as being distributed on a “wild–captive continuum” among the different situations [13]. Therefore, the issue of the likelihood of the results obtained on animals in a certain situation being also valid for those in a different one on the “wild-captive” spectrum needs careful scientific consideration. On one hand, the possibility to apply the results (e.g., in terms of issues that need investigating the most, and of parameters used for assessment) gathered from animals housed and managed in one condition to those in different conditions, would save the costs of conducting ad hoc research for each condition of the continuum. On the other hand, in ethology, there is usually not such a thing as a “golden standard” to be used to assess a criterion’s validity. Therefore, the risk of using measures that, although reliable, are not valid in the new condition is not to be underestimated.

Another important aspect of any measurement is its feasibility, that is, the practicality of the measure for use in the field [37], and feasibility is likely to be influenced by the management systems of the animals, as well. In order to give a basis to discuss external validity, a brief summary of the main welfare research on zoo African elephants will be given.

### Approaches to Zoo African Elephants’ Welfare Assessment

In 2002, Clubb and Mason [29] published the first comprehensive study on elephant welfare, highlighting the lack of agreement about the best practice and the absence of reference to welfare in the standards. Available data on elephant welfare indicators were collected through an extensive search of the literature and records of studbooks; communication with several organizations and scientists; and scientist’s visits to facilities. Variables which were likely to influence the welfare (general husbandry, social aspects, handling and training, and source of the animal) and four groups of welfare indicators for which data exist for zoo elephants (overall mortality rates, causes of mortality and morbidity, and reproductive and behavioral problems, including stereotypies and aggression directed to conspecifics or humans) were included. Zoo elephants were compared to elephants kept in extensive system (Asian timber camps) and wild populations, finding that only mortality significantly varied among the data available for both conditions [29,38].

In 2008, the final report of another comprehensive study was published by Harris et al. [33]. Opinions from an elephant welfare expert panel on the most important welfare indicators and factors of zoo/safari park elephants were gathered, and variables to collect data directly in the zoos were selected. The experts included zoo curators and managers, zoo elephant keepers, zoo vets, zoo inspectors, biologists, and behavior and welfare scientists. Data on husbandry, behavior (e.g., eating, drinking, social behavior, sleeping, excessive aggression, and stereotypies), health (especially musculoskeletal system: foot health score and locomotion score), a body condition score, and fecal cortisol metabolites were collected in 13 UK zoos and wildlife parks. Elephant history, keeper and health check questionnaires, and sheets for house and paddock descriptions were also used. The prevalence of health and behavioral indicators of poor welfare was described and it was possible to correlate space allowance with stereotypes and gait.

These studies notwithstanding, in 2010, Kreger and Hutchins [39] still noted that welfare guidelines for elephant management in zoos were mainly based on experience and not on scientific data and that they somewhat disagreed.

In 2013, Carlstead et al. [40] presented a project, which used an epidemiological approach to correlate the prevalence of positive and negative welfare states with the environmental, management, and husbandry factors that affect the welfare of zoo elephants. The animal-based indicators identified referred to behavior (e.g., walking distances, stereotypies, and laying behavior [41,42,43]), physiology (serum progesterone and prolactin [44]), and health (e.g., body condition score, abnormalities in the musculoskeletal system of the limbs, and foot physical examination [45,46]), and were based on seven animal-based criteria among the twelve in the Welfare Quality Protocol. The data were collected with different methods in American Zoo Association accredited Zoos: directly by on-site zoo personnel, retrospectively, and through surveys for experienced staff members. The results of the research project were published in 2016–2017 [41,42,43,44,45,46,47,48,49,50,51].

In 2015, a study commissioned by the Department of Environment, Food Rural Affairs (DEFRA), focused on the behavioral indicators as part of a wider set of indicators to assess the welfare of elephants in UK zoos [52]. Behavioral indicators and resources were identified through a literature review and focus group teleconferences with stakeholders, and were reviewed with the project’s External Advisory Panel. The 25 stakeholders included keepers, curators/managers, veterinarians, and zoo-based researchers working in 12 Ireland/UK zoos and 11 other, independent worldwide researchers studying captive and/or wild elephants’ behaviors. The panel selected 76 behavioral measures and grouped them into twelve categories (social interactions, abnormal behavior, arousal behavior, qualitative assessment, behavior occurring under stress, vocalizations, cognitive measures, environmental interactions, facial expressions, species-appropriate behaviors/activity budgets, defense behaviors, and comfort behavior/self-maintenance). The prototype tool consisted of three sections, the qualitative behavioral assessment, daytime behavior questions, and nigh-time behavior questions, and was designed to take no longer than 30 min to complete. A reliability, validity (i.e., inter-rater reliability and test re-test reliability, internal consistency, face validity, and construct validity testing predictions) and feasibility trial, conducted in three UK zoos, led to a further selection of the behavioral measures to be included in the tool. Further trials investigating reliability and validity lead to the development of a final evidence-based welfare assessment tool for zoo elephants, whose items had been selected based on their accuracy, validity, feasibility, and practicality of use by elephant keepers [53]. Using the same methodology, another project’s part provided evidence suggesting changes in the Secretary of State Standard Modern Zoo Practice guidelines for elephants [52].

## 3. Dedicated Protocols for Semi-Captive Elephants’ Welfare Assessment in South African Facilities Addressing the External Validity and Ethical Acceptability Issues

All the approaches for elephants’ welfare assessments described above have been developed for a target elephant subpopulation, mainly zoo animals, that differs from semi-captive elephants involved in interactive animal–visitor experiences in South Africa. Most of the approaches had undergone some form of reliability and validity testing, usually, intra or inter-rater concordance, as brilliantly reviewed by Williams et al. [14]. However, to our knowledge, the issue regarding whether the method developed could be suitable to be applied to other subpopulations of animals in a different management situation (i.e., external validity issue) was never investigated. An independent approach, taking into account the external validity issue, was initiated in 2013 by researchers of the University of Padua [54,55], as part of a running project including science, ethics, and education (of staff and tourists) and focusing on welfare, conservation, and their connections. The project included the proposal for two protocols, one for evaluating the welfare effects of the elephants’ general housing and management (i.e., housing and management protocol, HMP) and one for issues specifically related to the acceptability of interactions with visitors (i.e., animal–visitor interactions protocol, AVIP). Both protocols merge a scientific approach with ethical analysis (see Appendix A, Table A1 for details) in order to evaluate the overall welfare of the elephants kept in semi-captive conditions and involved in interactions with visitors in South Africa.

The proposed AVIP was adapted from a general animal–visitor interactions ethical evaluation protocol that was described elsewhere [21,56]. The HMP consisted of two procedures, one derived from the results obtained from animals in a different context (“external procedure”) and one based almost exclusively on results of preliminary ad hoc studies targeting the semi-captive, visitor interacting elephants under study (i.e., “internal procedure”), using different methods and paradigms [40,50,52,57,58,59,60,61,62,63,64] and then comparing their results (Appendix A, Table A1).

As can be seen from the very short description of the project in Appendix A, Table A1, a protocol for welfare assessment which explicitly addresses the issue of external validity and includes a procedure that is based uniquely on data collected on the target population can be very complex and demanding in terms of time and resources. Other ways to address the external validity issue when designing a project are possible. However, the need to generate data and validate the parameters within the target subpopulation, is likely to result in a demanding project in any case.

An alternative is to directly apply a protocol designed for zoo elephants to semi-captive interacting elephants and then try to assess its reliability and validity. However, the absence of a “golden standard” known external criterion [61,65] makes assessing welfare without conducting experiments to validate the imported parameters in the target population itself more likely to incur validity problems, which could be very difficult to be detected a posteriori. Therefore, caution should be used when exporting welfare assessing methods to other subpopulations than the one they have been designed for. More scientific research is needed to address the external validity issue in welfare assessment.

The idea that there could be specific welfare problems in different subpopulations of animals, and that preliminary data on which to develop a welfare assessment protocol should be collected on the target populations itself is suggested by Pritchard et al. [66] for working equines. However, to our knowledge, the issue of the external validity of protocols and indicators used to assess welfare in animals has not yet been explicitly addressed in the scientific literature. In July 2019, a search on Web of Science [67] using “external validity” and “welfare assessment” as keywords resulted in zero entries. However, recently some authors have begun expressing concerns on the possible limitations of applying a known validated welfare assessment protocol to farming systems differing from those for which it had been created [68,69]. Scott et al. [70] in their detailed review on quality of life assessment for humans and other animals does not discuss external validity among the important facets of validity to be addressed. This is surprising, because external validity is recognized to be an important facet of validity in other contexts (e.g., for canine temperament studies [71,72]). In biomedical research, the low external validity of the findings is acknowledged as a problem and there is scientific research on how to improve it (e.g., [73] on whether standardization of the living conditions increases or decreases external validity). The concern about the external validity of the findings extends to human welfare research (e.g., [74] on sampling methods to increase it).

## 4. Hints to the Existence of Relevant Differences in Welfare Evaluation between Zoo and Semi-Captive Elephants

The project initiated by Padua University was ambitious, and until now, alongside a range of educational activities, it was possible to complete the designing of the protocol AVIP and its application to the zoo context [21] and some preliminary studies [55]. The issues of external validity and ethical acceptability were explored in a questionnaire-based survey that involved experts [55,75].

### 4.1. Results for the External Validity Issue

Regarding the external validity issue, the questionnaire asked experts the following:

1. Which were the five most important topics for captive elephants (and the most relevant parameters to be used to evaluate them, as in Whay [60]?
2. Did the five most important elephant welfare topics identified have equal relevance for semi-captive compared to zoo-bound individuals?

Further sections asked experts to identify behavioral correlates of positive and negative emotions in elephants, questions on *stockmanship*, on health protocols to apply, and opinions on ethically relevant topics concerning captivity and involvement in activities with tourists. The questionnaire used for the study [55] is included in Appendix A as Table A2. Seventy-nine experts (defined as keepers, researchers, facilities managers, vets with at least five years of experience with African elephants, and government legislative authorities and animal rights activists dealing with African elephants) were contacted. Forty experts answered and twelve completed the survey on time, whose details are given in Appendix A, as Table A3. The results of the survey are detailed in Table 1 and Table 2.

Half of the experts stated that at least some of the issues they ranked as the five most important to captive elephants’ management had a different level of relevance for semi-captive individuals. Of the 55 answers given regarding equal relevance, 13 (23.6%) were negative answers. When the issues were grouped into 12 categories (i.e., social life, psychological alterations, foot pathologies, exhibit design, diet, musculoskeletal problems (including arthritis), environmental and behavioral enrichment (including occupational options), weight imbalances, training, use of chains, stability of the group, and reproductive management), eight categories (66.7%) were deemed to differ in relevance between zoo and semi-captive elephants by at least one expert. It is noteworthy that all the issues deemed of different relevance at least by some experts (e.g., musculoskeletal and foot health, diet and weight, and interaction with the environment) corresponded to parameters included as important in some of the previous literature about welfare assessments of zoo elephants [41,45,46,49,52,53]. The main differences stated by experts regarding the welfare topics are summarized in Table 2.

Although the abovementioned findings result from a pilot with relatively low sample size, they show that scientists deem the condition of semi-captive elephants different from that of zoos’ ones from a welfare assessment point of view. The results of this study, hence, suggest the existence of a possible external validity issue when using the same parameters and evaluation protocols in the two situations.

### 4.2. Results about the Ethical Acceptability of Procedures

When discussing the welfare conditions of semi-captive elephants in South Africa as being different from that of zoos’ ones, it is important to discuss also, the ethical acceptability of procedures that are typical of semi-captive animal–visitor interactions. As stated in [15,16], ethics play a role not only in deciding what level of welfare to assure to animals, but also in the decisions about which practices to accept or to avoid in managing each specific animal population. Welfare experts assessing the acceptability of practices are likely to be influenced by the context and culture they are part of, making their evaluation (of the practice) different for different subpopulations of animals. If this is the case, an experts’ evaluation of the acceptability of a practice can lead to that practice being either included or excluded from a management system. Because these different practices can influence welfare, their inclusion or exclusion is highly likely to influence the quality of life of the animals belonging to that subpopulation. Therefore, ethics is crucial to assess specific, controversial, welfare-relevant issues by focusing on their acceptability, which can vary depending on the animal population studied. Moreover, when assessing welfare, the level of welfare found in one specific situation must be compared to a standard threshold to determine whether acceptable or not. This level of acceptability, or the benchmark, will be determined by the findings of the evaluation procedure of experts and what they perceive as acceptable, thus highlighting the importance of the acceptability issue where welfare is concerned.

The survey in Appendix A, Table A2 included a section specifically investigating the ethical acceptability of procedures that are commonly applied with the semi-captive elephant subpopulation. The experts were asked to choose among three options: totally acceptable, partially acceptable, and totally unacceptable. In case experts chose the option “partially acceptable,” they were asked to specify under which conditions the procedure was acceptable. The list of procedures and results of the survey are detailed in Table 3.

It is noteworthy that there was no agreement among the experts on the acceptability of any of the procedures. This notwithstanding, there was more agreement in favor of the acceptability of some procedures, such as enrichment and training for medical practices. On the contrary, procedures such as chaining and “breaking in” were mostly seen as unacceptable. Among the more controversial procedures were those referring to interactions with tourists, including walking with tourists and training for shows: one expert deemed them totally acceptable, while others were equally divided between finding them partially acceptable and totally unacceptable. Additionally, free contact between elephants and people divided experts, the majority of them finding the procedure unacceptable.

It is interesting to note that one expert cited as a reason for the acceptability of free contact between elephants and people, it being part of the culture of the people involved. Free (i.e., unprotected) contact is a dangerous practice, which can often be seen in facilities offering “elephant experiences” to tourists in Southern Africa. Accidents happen, resulting in fatalities among the handlers (e.g., [76]). On the contrary, free contact with elephants is discouraged by zoo associations in western zoos, where protected contact is recommended [26,77,78]. In a sense, chaining, the use of punishment and/or of negative reinforcement, and the “breaking-in” procedure [29,76] to get young elephants used to being tame around people can be seen as a way to attempt decreasing the risks linked to unprotected contact [79]. The coercive form of breaking in is awfully cruel and does not deserve any discussion here [80]. Even the other procedures can be at high risk of being detrimental for the welfare of the animals involved; and they increase stress and frustration, thus increase the risks they are supposed to mitigate.

In the case of chaining, however, the way elephants are accustomed to wearing the chains, as well as the situation and the length of time they have to wear them and other variables, can influence the likelihood that the procedure has a welfare relevant effect on the animals. Whether you train an elephant to accept a chain or tether, or you use a chain or tether to train an elephant makes a huge difference in this discussion. Some people feel strongly that an elephant trained to take a chain is less stressed when the tether or chain is used during the application of medical procedures, thus creating safe options for veterinarians and the elephant. This brings to the fore the ethical consideration of whether welfare is compromised if an elephant is not desensitized/positively conditioned to any restraining device, whether a tether or chain or mechanical restraining device. For some people, tethering an elephant is acceptable also in other situations than medical procedures, provided that, due to appropriate training, the elephants have not developed a negative perception of the chain or tether. Of course, it is also important that the animals have a non-negative perception of the whole situation in which they are tethered, not only of being tethered per se. However, to what extent training a wild animal to be restrained is equally as acceptable as training a domestic one is open to debate. Moreover, the general aim for which animals are restrained or subjected to other management procedures is likely to be relevant when deciding on acceptability. On the other hand, there are people who are concerned by any form of restraint applied to a wild animal, independently from the method used to accustom the animal to it, from the aim of the restraint or even from the perception that the animal has of the situation.

For these reasons, the discussion of acceptability of procedures should be conducted on the level of each subpopulation of wild animals under investigation, and ethical arguments should not be easily exported from other contexts.

## 5. Conclusions

As wild animals could be subjected to a range of different management situations on the wild–captive continuum, ethical issues, scientific protocols, and indicators derived from studies conducted on animals on one position of the range (e.g., zoo animals) and found to be suitable for that subpopulation may not be suitable for other populations of animals of the same species. Given the lack of an external golden standard in welfare science, against which to verify whether the welfare assessment method used is indeed assessing the relevant aspects of welfare for those specific animals and all of them, the risk of not detecting a lack of suitability is high. If the method used to assess welfare is not valid, then the risk of not ensuring ethically acceptable levels of care and welfare for those animals is likely to be high. Experts appear to be aware that welfare issues that can be important for one subpopulation of animals may fail to be of equal importance for other subpopulations. For example, when asked about possible differences between zoo and semi-captive elephants, experts stated that semi-captive elephants were less likely to be affected by physical problems due to lack of exercise and restraint. They were also less likely to be affected by psychological ones derived from not being able to have access to an environment in which they could express their natural behavior and find suitable resources, at least for part of their time. However, differences between different subpopulations may not be confined only to likelihood of occurrences. For example, obese or lame elephants are likely to be rarer in semi-captive situations. However, if a semi-captive elephant is obese or lame, could he/she be more affected by his/her condition than a zoo one? Could he/she be more frustrated as she cannot take full advantage of the opportunity to roam free over a larger environment with the group he/she belongs to? Studies explicitly evaluating the differences, both in terms of likelihood of occurrence/prevalence (at the population level) and in terms of severity of effects at the individual level are needed. As Turner highlighted for extensive farm-animal breeding [81], focus should be directed also on assessing the availability, use, and success of contingencies preventing suffering and on *stockmanship*. It is evident that *stockmanship* becomes even more important in elephants involved in animal–visitor interactions (AVIs), and therefore, it should be investigated in more depth in this sub-population.

Moreover, the involvement of different animal subpopulations in management procedures whose ethical acceptability is controversial can vary both qualitatively and quantitatively. It is, therefore important for the scientific community to be aware and to discuss the issue of exporting findings and protocols developed in zoo animals to other populations that are managed differently. The approach suggested by this study can help to foster the role of experts in assessing the external validity and acceptability of procedures when exporting findings. In general, further interdisciplinary studies, like the one proposed by the AVIP protocol [21], are needed to investigate the complex issue of tourist interactions with animals in their differences along the “wild–captive continuum.” Further studies are needed as well to involve not only experts, but also the other stakeholders in the discussion about the issue of exporting findings along this continuum.

## 6. Patents

Independently from their points of view on the different situations in which animals can be found in the “wild–captive continuum,” and from the different regulations of practices involving wild animals in different countries, the authors of the present paper recognize the overriding importance of working together with all stakeholders and role-players to ensure that every effort is made to grant these wild animals the highest possible welfare standards.

## Figures and Tables

**Table 1:** Results of the survey regarding issues affecting the welfare of elephants in captivity and possible differences in relation to semi-captive individuals. E stands for whether the issue had equal relevance for semi-captive individuals compared to zoo ones; Y means that the experts answered it had equal relevance; and N means it had different relevance.

Expert	1st Issue	E	2nd Issue	E	3rd Issue	E	4th Issue	E	5th Issue	E
1	Social-environment	Y	Free of chain	N	Environmental Enrichment/Behavioral Enrichment	N	Food (and water) ad libitum	N	Breeding situation and Family management	Y
2	Social-environment	Y	Free of chains or other restricting measures	N	Environmental Enrichment/Behavioral Enrichment	N	Free choice of food given throughout the day	N	Proving a breeding possibilities and family life	Y
3	Foot disease	N	Arthritis	N	Psychological distress	N	Social-Environment	N	Weight imbalance: overweight or underweight	N
4	Health	Y	Behavior	Y	Social structure	Y	Facilities	Y	Management	Y
5	Social-environment	Y	Environment	Y	Training relationship	Y	Enrichment	Y	Occupational options	Y
6	Negative Affective States	Y	High stereotypic behavior rates	Y	Social-environment	Y	Hormone Imbalance	Y	Foot and Joint Health	Y
7	Foot health	Y	Musculo-skeletal health	Y	Nutrition	Y	Behavioral/Enrichment	N	Husbandry training	Y
8	Access to water and food	Y	Adequate space and safe housing	Y	Training, positive and negative reinforcement	Y	Health	Y	Social-environment	Na
9	Psychological alterations	Y	Na		Na		Na		Na	
10	Mental behavioral health	Y	Social-environment	Y	Physical Health/physical well being	Y	Foot condition	Y	Space and exhibit design	Y
11	Freedom from thirst and hunger	Y	Freedom of shelter	Y	Freedom from pain, injury, and disease	Y	Freedom to express species-specific behavior	Y	Freedom from fear and distress	Y
12	Musculo-skeletal disorders	Y	Foot disease	Y	Obesity	N	Injuries/stress due to the inadequate social environment	Y	Stereotypic behavior	Y

**Table 2:** Differences stated by experts between zoo elephants and semi-captive ones regarding main welfare relevant topics.

Expert	Stated Differences Regarding Welfare Indicators in Semi-Captive Elephants as Compared to Zoo Ones
1	Issue n°2 (free of chains) and n°3 (enrichment) are less important for semi-captive elephants, whereas issue n°5 (breeding/Family management) is more relevant than for zoo elephants. Issue n°4 (food (and water) ad libitum) less relevant for semi-captive elephants because they can search for them when free to roam.
2	Issue n°2 (free of chains) is less of a problem for semi-captive elephants because if they are chained, they are usually chained for shorter periods, usually at night. Issue n°3 (enrichment) and issue n°4 (free choice of food) are less important for semi-captive elephants because, for a part of the day they are free to roam and forage in a larger environment where they can express their natural behavior.
3	Issue n°1 (foot disease) and issue n°2 (arthritis) are less relevant for semi-captive animals because their movement is less restricted. Issue n°3 (psychological distress) is less relevant for semi-captive elephants because they are less deprived than zoo ones. Issue n°4 (social alienation or isolation) and issue n°5 (weight imbalance) are more common in a strictly captive setting, although their relevance for the affected animal is the same in both contexts.
7	Issue n°4 (behavioural/enrichment) is stated to differ, but no further explanation is given.
12	Issue n°3 (obesity) is less common in a semi-captive setting, although its relevance for the affected animal is the same in both contexts.

**Table 3:** Results of the acceptability survey.

Management Practice	Totally Acceptable	Partially Acceptable	Totally Unacceptable	Notes and Main Specifications Given
Free contact	2	3	5	2 missing answers; if the animal needs medical care/never for medical interventions/never for aggressive, nervous elephants or elephants in musth; it is difficult to eradicate, it is cultural
Interaction with tourists	1	4	4	3 missing answers; only if there is a barrier and tourists receive an education. Only elephants with the right disposition, with some training. Trained with positive reinforcement methods
Chaining during riding	0	2	7	3 missing answers; when elephants are free to roam it is acceptable when tourists get on and off
Walking with tourists	1	4	4	3 missing answers; only if there is a barrier, only if there is a trainer, only if they only walk side by side, only if elephants can walk off when the walk is finished
Enrichment	8	1	0	3 missing answers; acceptable only if not used in place of granting the elephants the necessary freedoms
Training for medical procedures	8	2	0	2 missing answers; positive reinforcement methods only, protected contact
Training for shows	1	4	4	2 missing answers; only if the behaviors trained are natural behaviors, only if there is an educational goal, elephants must be monitored
Training as enrichment	4	5	0	2 missing answers; only if it is not the only form of enrichment, only positive reinforcement methods, repetition of already learned behaviors is not enriching, acquiring new ones is likely to be
Training with negative reinforcement	1	3	5	3 missing answers; only to stop dangerous behavior, limited holds are ok, should be monitored
“Breaking in” methods	1	0	7	3 missing answers; 1 no opinion

## Appendix A

**Table A1:** Summary of the main methods and steps included in the project for the housing and management protocol (HMP).

Step	Methodological Approaches	Specific Studies (If >1)	Brief Description and Relevant References	Reliability/Validity Checking
Identifying parameters to be included in the tool using an internal procedure (IP)	Consensus procedures—Delphi and Ethical Delphi (CP)	Stakeholders Consensus (SC)	Analogously to what has been done by Gurusamy et al. [58] and by Chadwick et al. [59], the point of view of stakeholders will be collected. Stakeholders will be asked to rate the effect of possible welfare issues, identified during a pilot study, on both single elephants and the population, and a Delphi procedure will be used to approach consensus.	Consensus among participants.
		Expert Consensus (EC)	Following the classical Delphi and the Ethical Delphi methods (e.g., [60,61,62]), each expert in the panel is asked to: (a) rate risk factors and issues, which could affect the welfare state of captive elephants; (b) identify relevant measures, including both bad and good welfare criteria, and possible relevant differences for “semi-captive” individuals.	Consensus among participants.
	Experimental approach validating behavioral correlates of positive and negative mental states in elephants (EA)	Emotional valence study	Detailed analysis of the behavior (as in Young et al. [63]), expressed by elephants during specific situations, whose emotional value has already been experimentally established (using avoidance or motivation paradigms), is used to identify behavioral correlates of positive and negative mental states). Physiological parameters (such as salivary cortisol) are also evaluated.	Intra and inter-observer reliabilities checked for the behavioral observations. 30% of the videos also analyzed by a blind observer, and qualitatively assessed by experts.
		Study on anticipatory behaviour	As in Clegg et al. [64].	Intra and inter-observer reliability checked for the behavioral observations.
	Correlational Study (CS)		Data on feeding, freedom of movement, physical comfort, health status, appropriate social/non-social behavior, human-elephant interactions and *stockmanship*, avoidance of negative and presence of positive emotions, control over the environment gathered using structured interviews, ad hoc developed questionnaires, direct observation, quantitative and qualitative videotaped behavioral observation, analysis of cortisol concentrations in suitable matrices, and health evaluation (clinical visit and medical and reproductive entries) for all South African semi-captive elephants. Correlations between income measures (e.g., characteristics of the facilities) and outcome measures (e.g., behavioral signs of negative or positive psycho-physical states), are statistically investigated as in Carlstead et al. [40]. A genetic study of the captive elephant population also planned.	Inter-observer and test-retest reliabilities checked. Videos analyzed (qualitatively and quantitatively) also by blind observers and qualitatively by experts.
Unifying IP parameters			EC + EA results are compared and used to interpret CS results. The valence to be attributed to behavioral outputs recorded in CS is identified using EC + EA results.	
Identifying parameters to be included in the tool using an external procedure (EP)	Welfare Quality-based “Elewell” (EW)		After a detailed literature review (e.g., [50,52]) and expert opinion seeking on the target species, an approach similar to the European Welfare Quality^®^ project for some domestic species was developed. It has then been applied to semi-captive elephants in a pilot study.	Inter-observer intra-observer (on videos) and test-retest reliabilities checked.
Verifying the chosen parameters	Comparing IP and EP results		IP and EP results in terms of welfare parameters will be compared in order to investigate the biological validity issue.	External validity issue tackled.
	Cognitive bias paradigm (CB)		A cognitive bias paradigm [57] experiment performed on two groups of elephants: eight elephants resulted from IP + EP to have highest welfare levels vs eight elephants (similar in temperament, gender, age, history) found to have the lowest. The expected result is to find a pessimistic bias in the elephants found to have the lowest levels.	
Draft			Protocol draft created using the parameters found to be feasible, suitable, reliable and valid.	
	Stakeholders and Role-players Discussion (SRD)—Ethical Matrix		Discussion among all stakeholders and role-players in a workshop using the Ethical Matrix tool, in order to reach consensus on weighing the parameters and establishing a minimum acceptability threshold (the results of the protocol represent the stakeholder “elephants”).	
Tool			On facility welfare assessment tool created.	

**Table A2:** Questionnaire. It was designed and used by the University of Padua team for the cited expert opinion survey [55,75].

**1. Welfare issue n°1 to be assessed:**________________________________________________________________	
**2. How important is welfare issue n°1 to each individual animal in the context of your expertise?** (0 = minimum; 5 = maximum) *Score: 0 1 2 3 4 5* *If you can, explain the reason of choosing this score:*_____________________________________________________	
**3. How important is welfare issue n°1 to the captive group of animal in your context?** (0 = minimum; 5 = maximum) *Score: 0 1 2 3 4 5* *If you can, explain the reason of choosing this score:*_____________________________________________________	
**4. Is welfare issue n°1 equally relevant in semi-captive context?** ☐ Yes, always ☐ No **If it doesn’t, which is more important?** _______________________________________________________	
**5. What measures do you think are useful indicators of this welfare issue?** Please give brief methodological details.	**6. How is this measure important to indicate the issue?** (0 = minimum; 5 = maximum)
*Measure n°1:* _______________________	*Score: 0 1 2 3 4 5*
*Brief details:* _______________________	*Comments:* _______________________
*Reference (if possible):* _______________________	
*Measure n°2:* ________________________	*Score: 0 1 2 3 4 5*
*Brief details:* _______________________	*Comments:* _______________________
*Reference (if possible):* _______________________	
*Measure n°3:* ______________________________	*Score: 0 1 2 3 4 5*
*Brief details:* _______________________	*Comments:* _______________________
*Reference (if possible):* _______________________	
The above template was repeated other four times for other four welfare issues the expert could identify as the second to fifth most important.	
**31. In your opinion and within the context of your situation, how do African elephants express positive emotions?** ______________________________________________________________________________________________________________ ______________________________________________________________________________________________________________ ______________________________________________________________________________________________________________	
**32. Among the ways elephants express positive emotions in context you described, which are the 3 most useful indicators to use in order to assess welfare in practice?** Please describe only the situation of your context. Elephant alone: _____________________________________________________________________________________ ☐ I don’t have this kind of experience Elephant in herd: ___________________________________________________________________________________ ☐ I don’t have this kind of experience Elephant in wild: ___________________________________________________________________________________ ☐ I don’t have this kind of experience	
**33. In your opinion and within the context of your situation, how do African elephants express negative emotions?** ________________________________________________________________________________________________________________ ________________________________________________________________________________________________________________ ________________________________________________________________________________________________________________	
**34. Among the ways elephants express negative emotions in context you described, which are the 3 most useful indicators to use in order to assess welfare in practice?** Please describe only the situation of your context. 4. Elephant alone: _______________________________________________________________ ☐ I don’t have this kind of experience 5. Elephant in herd: ______________________________________________________________ ☐ I don’t have this kind of experience 6. Elephant in wild: ______________________________________________________________ ☐ I don’t have this kind of experience	
**35. On a scale from 1 to 100, how much does *stockmanship* affect the welfare of captive African elephants?** **in trained animals:** _______ ○ Protected contact: _______ ○ Free contact: _______ ○ No contact: _______ ○ Confined contact: _______ **in untrained animals:** _______	
**36. In your opinion, are there useful matters in order to evaluate welfare aspects specifically related to *stockmanship* in captive African Elephants?** Please list some related measures. **in trained animals:** ○ **Matter A1:** ______________________________________________________________________________ ▪ *Measure 1:* _______________________________________________________________________ ▪ *Measure 2*: _______________________________________________________________________ ▪ *Measure 3:* _______________________________________________________________________ ○ **Matter B1:** ______________________________________________________________________________ ▪ *Measure 1:* _______________________________________________________________________ ▪ *Measure 2:* _______________________________________________________________________ ▪ *Measure 3:* _______________________________________________________________________ **in not trained animals:** ○ **Matter A2:** ______________________________________________________________________________ ▪ *Measure 1:* _______________________________________________________________________ ▪ *Measure 2:* _______________________________________________________________________ ▪ *Measure 3:* _______________________________________________________________________	
**37. In your opinion, list the 3 most important factors that affect the way stockman treats animals.** 1) _____________________________________________________________________________________________ 2) _____________________________________________________________________________________________ 3) _____________________________________________________________________________________________	
**38. List the 3 most important aspects for good captive African elephants *stockmanship*?**	**39. How much would this aspect improve the welfare of the African elephants?** (0 = minimum; 5 = maximum)
***Aspect n°1:*** __________________________________________	*Score: 0 1 2 3 4 5*
	*Comments:*
***Aspect n°2:*** ____________________________________________	*Score: 0 1 2 3 4 5*
	*Comments:*
***Aspect n°3:*** ___________________________________________	*Score: 0 1 2 3 4 5*
	*Comments:*
**39. How do you consider the following situations about elephants in semi-captive condition?** **Free contact with handler** ○ Totally acceptable ○ Partially acceptable (please write conditions _______________________________________________) ○ Unacceptable ○ I have no opinion **Training for medical procedures** ○ Totally acceptable ○ Partially acceptable (please write conditions _______________________________________________) ○ Unacceptable ○ I have no opinion **Training for show** ○ Totally acceptable ○ Partially acceptable (please write conditions _______________________________________________) ○ Unacceptable ○ I have no opinion **Training as enrichment** ○ Totally acceptable ○ Partially acceptable (please write conditions _______________________________________________) ○ Unacceptable ○ I have no opinion **Training with negative reinforcement** ○ Totally acceptable ○ Partially acceptable (please write conditions _______________________________________________) ○ Unacceptable ○ I have no opinion **Breaking method to train the elephant** ○ Totally acceptable ○ Partially acceptable ○ Unacceptable ○ I have no opinion	
**40. Please, feel free to give us some further comments or advices on assessing welfare in African elephants in captivity:** _________________________________________________________________________________________________________ _________________________________________________________________________________________________________ _________________________________________________________________________________________________________	
**HEALTH PROTOCOL.**	
**41. In your opinion, how important is regular health checks protocol for the welfare of captive African elephants?** (0 = no importance; 10 = fundamental) *Score: 0 1 2 3 4 5 6 7 8 9 10*	
**42. Which are the six most important clinic/diagnostic procedures that should be adopted in order to assess the health status of African elephants in captivity?**	
***Measure n°1***: ______________________________________	***Measure n°4***: ______________________________________
*Brief detail:*	*Brief detail:*
*Is it possible to perform the procedure on:* trained animals?: ○ Yes, only during sedation ○ Yes, without sedation ○ No untrained animals?: ○ Yes, only during sedation ○ Yes, without sedation ○ No	*Is it possible to perform the procedure on:* trained animals?: ○ Yes, only during sedation ○ Yes, without sedation ○ No untrained animals?: ○ Yes, only during sedation ○ Yes, without sedation ○ No
*How often should it be repeated?*	*How often should it be repeated?*
***Measure n°2:*** ________________________________________	***Measure n°5:*** ________________________________________
*Brief detail:*	*Brief detail:*
*Is it possible to perform the procedure on:* *trained animals?:* ○ Yes, only during sedation ○ Yes, without sedation ○ No *not trained* animals?: ○ Yes, only during sedation ○ Yes, without sedation ○ No	*Is it possible to perform the procedure on:* *trained animals?:* ○ Yes, only during sedation ○ Yes, without sedation ○ No *not trained* animals?: ○ Yes, only during sedation ○ Yes, without sedation ○ No
*How often should it be repeated?*	*How often should it be repeated?*
***Measure n°3:*** _______________________________________	***Measure n°6:*** ________________________________________
*Brief detail:*	*Brief detail:*
*Is it possible to perform the procedure on:* trained animals?: ○ Yes, only during sedation ○ Yes, without sedation ○ No untrained animals?: ○ Yes, only during sedation ○ Yes, without sedation ○ No	*Is it possible to perform the procedure on:* trained animals?: ○ Yes, only during sedation ○ Yes, without sedation ○ No untrained animals?: ○ Yes, only during sedation ○ Yes, without sedation ○ No
*How often should it be repeated?*	*How often should it be repeated?*
**43. Which are the six most important preventive procedures that should be adopted in order to prevent diseases/health problems of captive African elephants?**	
***Measure n°1***: __________________________________	***Measure n°4***: _______________________________________
*Brief detail:*	*Brief detail:*
*Is it possible to perform the procedure on:* trained animals?: ○ Yes, only during sedation ○ Yes, without sedation ○ No untrained animals?: ○ Yes, only during sedation ○ Yes, without sedation ○ No	*Is it possible to perform the procedure on:* trained animals?: ○ Yes, only during sedation ○ Yes, without sedation ○ No untrained animals?: ○ Yes, only during sedation ○ Yes, without sedation ○ No
*How often should it be repeated?*	*How often should it be repeated?*
***Measure n°2***: __________________________________	***Measure n°5:*** __________________________________________
*Brief detail:*	*Brief detail:*
*Is it possible to perform the procedure on:* trained animals?: ○ Yes, only during sedation ○ Yes, without sedation ○ No untrained animals?: ○ Yes, only during sedation ○ Yes, without sedation ○ No	*Is it possible to perform the procedure on:* trained animals?: ○ Yes, only during sedation ○ Yes, without sedation ○ No untrained animals?: ○ Yes, only during sedation ○ Yes, without sedation ○ No
*How often should it be repeated?*	*How often should it be repeated?*
***Measure n°3***: ___________________________________	***Measure n°6***: _______________________________________
*Brief detail:*	*Brief detail:*
*Is it possible to perform the procedure on:* trained animals?: ○ Yes, only during sedation ○ Yes, without sedation ○ No untrained animals?: ○ Yes, only during sedation ○ Yes, without sedation ○ No	*Is it possible to perform the procedure on:* trained animals?: ○ Yes, only during sedation ○ Yes, without sedation ○ No untrained animals?: ○ Yes, only during sedation ○ Yes, without sedation ○ No
*How often should it be repeated?*	*How often should it be repeated?*

**Table A3:** Experts’ demographic characteristics.

Expert	Sex	Age Category (Years)	Professional Involvement	Education	Continent	Area of Expertise on Elephants		
						Species	Situation	Training Status
1	M	30–40	Elephant trainer, keeper and scientific consultant	MSc Biology	Europe	Asian and African	Captive (zoo, encampment)	Both trained and untrained
2	F	Over 50	Scientist, scientific advisor	MSc, PhD	Europe	Asian and African	Wild, Semi-captive, Captive	Both trained and untrained
3	F	20–30	Wildlife Vet	B.S. Biological science, D.V.M. degree	Americas	Asian and African	Semi-captive, Captive	Trained
4	M	40–50	Vet, Elephant Supervisor and chief of animal behavioral Management, training consultant	D.V.M. degree	Americas	Asian and African	Semi-captive, Captive	Both trained and untrained
5	F	Over 50	Advocacy	Juris Doctor	Americas	-	Captive	Not answered
6	M	30–40	Animal Welfare Consultant	MS, PhD	Americas	Asian and African	Semi-captive, Captive	Both trained and untrained
7	F	Over 50	Wildlife Vet, university researcher	DVM, MS, MPH, PhD	Americas	Asian and African	Wild, Captive	Both trained and untrained
8	M	Over 50	Animal Scientist	BSc(Agric) Animal Production; BSc Hons Agric (Physiology); MSc (Agric) Reproductive physiology	Africa	African	Semi-captive, Captive	Both trained and untrained
9	M	40–50	Owner/head Keeper	-	Europe	Asian and African	Captive	Both trained and untrained
10	M	40–50	Scientific consultant	Masters	Americas		Wild, Captive	Both trained and untrained
11	F	-	Zookeeper/Elephant care specialist	Master	-	-	[Captive] -	-
12	F	Over 50	Director of Science, Research and Advocacy	Master of Science, Anthrozoology, Canisius College	Americas	Asian and African	Captive	Trained
